# Iodine Status of Infants and Toddlers under 2 Years of Age and Its Association with Feeding Behaviors and Maternal Iodine Status in Shanghai: A Quantile Regression Analysis

**DOI:** 10.3390/nu16111686

**Published:** 2024-05-29

**Authors:** Wei Zhou, Jingyi Si, Xue Han, Weiwei Zheng, Xiangting Li, Changfeng Zhu, Jiajie Zang

**Affiliations:** 1 Department of School and Nutrition, Shanghai Yangpu District Center for Disease Control and Prevention, Shanghai 200090, China; 2 Department of Gastroenterology and Hepatology, Zhongshan Hospital, Fudan University, Shanghai 200032, China; 16301050251@fudan.edu.cn (J.S.); zhuchangfeng@fudan.edu.cn (C.Z.); 3 General Office, Shanghai Yangpu District Center for Disease Control and Prevention, Shanghai 200090, China; 4 Division of Health Risk Factors Monitoring and Control, Shanghai Municipal Center for Disease Control and Prevention, Shanghai 200336, China

**Keywords:** urinary iodine concentration, iodine status, feeding behaviors, quantile regression

## Abstract

It is crucial to provide adequate iodine nutrition to infants and toddlers for proper thyroid function and subsequent brain development. Infants are particularly vulnerable to iodine deficiency during the transition from a milk-based diet (breast milk and/or infant formula) to solid food. This study examines the current iodine levels of children during their first two years of life and investigates the association between these levels and feeding behaviors and the iodine status of their mothers in Shanghai, a city located in eastern China. A hospital-based cohort study was conducted to enroll mother–child pairs, where the child is aged 6–23 months, who visited community health service centers in the 16 districts of Shanghai, China. Data on socio-demographic factors and feeding behavior data were collected from the participants. The urinary iodine concentration (UIC) in both the young children and their mothers were analyzed. A total of 2282 mother–child pairs were included in this analysis. The median (p25–p75) UIC for lactating women, weaning women, and children were 121.3 μg/L (68.1–206.4 μg/L), 123.4 μg/L (58.4–227.2 μg/L), and 152.1 μg/L (75.8–268.3 μg/L), respectively. The UIC in children was found to be higher than that in their mothers (*p* < 0.001). Children who consumed less than 500 mL per day of formula milk in the last week had lower UICs compared with those who consumed 500 mL per day or more (*p* = 0.026). Furthermore, the children’s UIC was positively correlated with the maternal UIC (*r_s_* = 0.285, *p* < 0.001). Multiple quantile regression analysis revealed a statistically significant positive association between maternal UIC and children’s UIC between the 0.1 and 0.9 quantiles (all *p* < 0.001). We found that the iodine status of infants and toddlers, as well as of mothers, was sufficient. However, a large minority of children and mothers may be at risk of iodine deficiency. Furthermore, no associations between children’s UIC and feeding behaviors were observed. Moreover, there was a positive correlation between the UIC of young children and their mothers.

## 1. Introduction

Iodine is essential for the synthesis of thyroid hormones [1]. Severe iodine deficiency during pregnancy is well known for potentially causing impaired brain development in the child, affecting cognitive and motor function, as well as hearing and speech [2]. Maternal thyroid hormone deficiency has been shown to alter the offspring’s brain structure through incorrect neuronal migration [3]. During the first 1000 days of life, these hormones are necessary for the myelination of the central nervous system, which is important for proper cognitive function. Inadequate supply of thyroid hormones during this critical period can also lead to irreversible mental retardation or cretinism [4]. Very high levels of iodide in the circulation, applied, for example, by iodine-containing ointment or lotion to the skin to infants, can promptly block thyroid hormone release from the thyroid gland and cause iatrogenic hypothyroidism. Transient hypothyroidism can also cause adverse neurodevelopmental outcomes [5]. Furthermore, iodine is also necessary for normal growth and bone maturation in children. It supports the development of healthy bones and contributes to overall physical growth and maturation [6]. Adequate iodine intake is therefore important for both cognitive and physical development in infants and children.

Iodine deficiency (ID) in the fetus and infant is the most common cause of preventable brain damage globally [2,7]. A systematic review from 2013 on the effect of ID on mental development in children 5 years and younger showed that even mild ID can influence the school performance, intellectual ability, and work capacity of children [8]. However, a recent review concluded that there is insufficient evidence to support recommendations for iodine supplementation in areas of mild or moderate iodine deficiency [9]. Despite the role of iodine in growth and development [10], we have little knowledge of young children’s iodine status in Shanghai.

A large part of the absorbed dietary iodine is eventually excreted in urine, primarily as iodine [11]. Ideally, 24 h urine collections should be obtained, but as compliance may be low for 24 h collections, estimates based on spot urine samples are often preferred [12]. Iodine excretion is then expressed as the median urinary iodine concentration (UIC) in the samples. The World Health Organization (WHO) advises the evaluation of the iodine status of a population by using the iodine concentration of a random spot urine specimen [13]. Urinary iodine only reflects iodine intake within the past few days, and as a clinical biomarker, it generally is not useful for classifying intake sufficiency or deficiency in a person; rather, it is used to define the risk of a population. Individual iodine status assessment methods are still in the exploration phase [14]. Thus, we explored using UIC to evaluate the iodine status of mothers and children in our study.

A previous study shows that two billion people worldwide, including 31.5% of children of school age, have insufficient iodine intake [15]. ID has been drastically reduced since the introduction of salt iodization programs in China; a cross-sectional study conducted across all 31 provinces of mainland China that included 78,740 participants aged 18 years or older has shown that the iodine nutrition level in the general population is within the safe range [16]. However, mild ID appears to have partially re-emerged in coastal areas of China because of the tendency to use non-iodized salt [17,18]. According to a recent study, the median UIC of pregnant women across China is 146 μg/L, which is close to the cutoff value of 150 μg/L [19]. Furthermore, previous studies have shown that pregnant women in Shanghai have mild ID [20]. In 2018, a study including 257 lactating women and their infants at 42 days postpartum showed that the iodine status of lactating women and infants in Shanghai was generally sufficient according to the WHO’s iodine nutritional status recommendation [21]. Whether infants are also iodine sufficient when they grow up to two years old is worth further investigation.

The WHO and the United Nations International Children’s Emergency Fund (UNICEF) recommend exclusive breastfeeding within 6 months after birth and continued breastfeeding for two years or more, together with safe, nutritionally adequate, age-appropriate, responsive complementary feeding starting in the sixth month [22]. The transition from a diet of breast milk and/or infant formula to a diet that includes solid foods and other beverages during infancy can lead to paramount changes in the intake of certain nutrients; hence, it is important to ensure the nutritional adequacy of complementary foods for infants. It is recommended that as infants are being introduced to complementary foods, supplementary breastfeeding should be continued until 2 years of age to cater for the infants’ iodine requirements [23].

For specific population groups, including infants and toddlers under 2 years old and their mothers, data regarding the iodine status of children and mothers are rare. To date, little has been reported on the feeding behaviors of infants and toddlers and the impact on their iodine status in China. Therefore, the purpose of our study was to evaluate the most updated and complete information on the iodine status of this specific population and to investigate the relationship between the iodine status of infants and toddlers and feeding behaviors, as well as with the maternal iodine status.

## 2. Materials and Methods

### 2.1. Study Population

We analyzed the data from the Iodine Status in Pregnancy and Offspring Health Cohort (ISPOHC) study, an ongoing and observational mother–child pair cohort study conducted by the Shanghai Municipal Center for Disease Control and Prevention. The study has already completed two rounds of data collection from 2017 to 2019.

Briefly, pregnant women who had lived in Shanghai for at least six months in the past year and who had established a healthy record at the local community health service center during different trimesters (first trimester ≤12 gestational weeks (GW), the second trimester (13–27 GW), and the third trimester (≥28 GW)) were recruited. The inclusion criteria for all pregnant women and detailed information about the study cohort, the study sites, and characteristics of the study group have been previously reported [24,25]. The first round of data collection took place from April 2017 to October 2017, during which pregnant women were enrolled in the study. After 42 days postpartum, mother–child pairs were followed-up. The second round of data collection occurred at least 6 months after childbirth, from March 2018 to June 2019. We collected data from 2282 mother–child pairs who met the criteria of having a singleton pregnancy and where both mother and child had provided urine samples during the second follow-up.

All participating mothers provided informed consent, and the study protocols were approved by the Ethics Committee of the Shanghai Municipal Center for Disease Control and Prevention (EC No. 2017/13, approval date: 25 April 2017).

### 2.2. Data Collection and Measurements

During the ISPOHC study, all mother–child pairs completed a face-to-face questionnaire to collect data regarding maternal basic information such as socioeconomic status and weight, as well as physical activity levels. The questionnaire also collected data on the feeding knowledge and behavior of the mothers, as well as the growth and development, feeding status, and nutrient supplement use of the young children. To assess the dietary intake of infants and toddlers, a well-designed food-frequency questionnaire (FFQ) was used.

Additionally, anthropometric measurements were taken for the infants and toddlers using standard procedures. Body weight was measured to the nearest 0.1 kg using an electronic scale with light clothes on (Seca, Hamburg, Germany). Height was measured to the nearest 0.1 cm using a horizontal length measuring instrument without shoes (Seca, Hamburg, Germany). Head circumference was measured at a 0.1 cm threshold using an inelastic tape measure. The WHO Anthro software (offline SPSS version (Windows)) [26] was used to calculate the Z-score for each child.

### 2.3. Analysis of Iodine Status

On the day of the visit, 5 mL of spot urine samples from mothers and children were collected into clean, tightly capped containers. The samples were stored at −80 degrees Celsius until analysis. The acid digestion method (As3 + −Ce4 + catalytic spectrophotometry) was applied to test each urinary sample in the Shanghai Municipal Center for Disease Control and Prevention [27]. Results of the iodine status are reported as the UIC (μg/L) for each mother–child pair. The UIC is considered an effective biochemical indicator for assessing iodine status at the population level, based on the epidemiological criteria recommended by the WHO, which also suggests that the median value for the sampled population is the most commonly assessed indicator. For non-breastfeeding women, iodine nutritional status is insufficient when the median UIC is less than 100 μg/L, adequate when the median UIC is between 100 and 199 μg/L, above requirements when the median UIC is between 200 and 299 μg/L, and excessive when the median UIC is above 300 μg/L. In lactating women and young children aged 2 years old or below, a median UIC of less than 100 µg/L indicates iodine insufficiency [28].

### 2.4. Definitions and Classifications of Relevant Indicators

In our study, we classified the current age of mothers into four categories: <29, 30–34, 35–39, and ≥40 years. Maternal education was divided into three categories based on the length of education: junior high school or below (≤9 years), high school or junior college (10–15 years), and university or higher (≥16 years). Total annual household income was divided into three types: low (CNY 0–149,000), medium (CNY 150,000–299,999), and high (≥ CNY 300,000). The self-reported height and weight of the mothers were used to calculate their body mass index (BMI) in kg/m^2^. BMI was then divided into four types according to WHO criteria: underweight (BMI < 18.5 kg/m^2^), normal weight (18.5–24.9 kg/m^2^), overweight (25–29.9 kg/m^2^), and obesity (≥30 kg/m^2^) [29]. The addition of salt to the infants’ food was classified into “iodized salt” or “never or non-iodized salt”. Smoking status and alcohol intake during lactation were classified into two categories: “yes” or “no”.

### 2.5. Statistical Analysis

Data processing and analysis were conducted using IBM SPSS Statistics version 27 (IBM Corp., Armonk, NY, USA). The normality of the data distribution was tested by using the Kolmogorov–Smirnov test. Normally distributed data are presented as the mean and standard deviation, while non-normally distributed data are presented as the median and 25–75th percentiles (p25–p75) as appropriate. Variable distribution was assessed through visual inspection of plots such as histograms and box plots. The Mann–Whitney *U* test or Kruskal–Wallis tests were used to assess the differences in UIC between different groups based on various characteristics. Spearman’s correlation analysis was conducted to examine the correlations between infants’ and toddlers’ UIC values and potentially related variables. A simultaneous quantile regression model was performed with children’s UIC as the dependent variable. The independent variables included maternal UIC, maternal BMI, maternal age, maternal educational level, children’s gender, children’s age, household income, children’s salt category, iodine supplement usage, feeding practice, and daily consumption of formula milk in the last week. The level of significance was set at *p* < 0.05.

## 3. Results

### 3.1. Characteristics of Infants and Toddlers and Their Mothers

The characteristics of infants and toddlers and their mothers participating in the second-round study are presented in Table 1. A total of 2282 mother–child pairs were included in this analysis. The mean (±SD) age of the mothers was 30.5 ± 4.4 years. The percentages of mothers aged <29, 30–34, 35–39, and ≥40 years were 45.3%, 34.9%, 16.7%, and 3.1%, respectively. Among all mothers, 75.4% had a normal weight. In terms of education, 38.8% of the mothers had a university degree or above. The majority of the mothers (61.3%) were primiparous. Nearly all the mothers (99.0%) were non-smokers, and 99.4% reported never drinking alcohol during lactation. Only 14.5% of the mothers used supplements, while 1.1% used iodine-containing supplements within a year.

The gender distribution among infants and toddlers was almost even, with 52.7% boys and 47.3% girls. Young children aged 6–11 months and 12–23 months accounted for 69.7% and 30.2% of the participants, respectively. The median (p25–p75) gestational age at birth was 39 weeks (38–40 weeks). Based on the weight-for-length/height z-score (WL/HZ), 0.7% of the infants and toddlers were wasted, while 7.1% were overweight. Furthermore, based on the BMI-for-age z-score (BAZ), 0.9% were underweight and 7.1% were overweight. The total annual household income is detailed in Table 1.

### 3.2. Iodine Status of Young Children and Mothers

The median (p25–p75) UIC of the infants and toddlers was 152.1 μg/L (75.8–268.3 μg/L), which met the WHO criterion for iodine sufficiency (>100 μg/L). The median (p25–p75) UICs of boys and girls were 148.7 μg/L (72.8–261.9 μg/L) and 155.3 μg/L (79.7–279.5 μg/L), respectively. Among the infants aged 6–11 months and toddlers aged 12–23 months, the median (p25–p75) UICs were 152.1 μg/L (74.2–273.9 μg/L) and 152.1 μg/L (83.6–250.8 μg/L), respectively. Table 1 presents the UIC values among the young children based on different characteristics. There were no significant differences in children’s UIC values between the different genders, age groups, WL/HZ, BAZ, and total annual household income levels. The median (p25–p75) UICs of lactating and weaning women were 121.3 μg/L (68.1–206.4 μg/L) and 123.4 μg/L (58.4–227.2 μg/L), respectively, both meeting the criterion for iodine sufficiency (>100 μg/L and 100–199 μg/L). There were also no significant differences in children’s UIC values between the different maternal characteristics.

**Figure 1:**
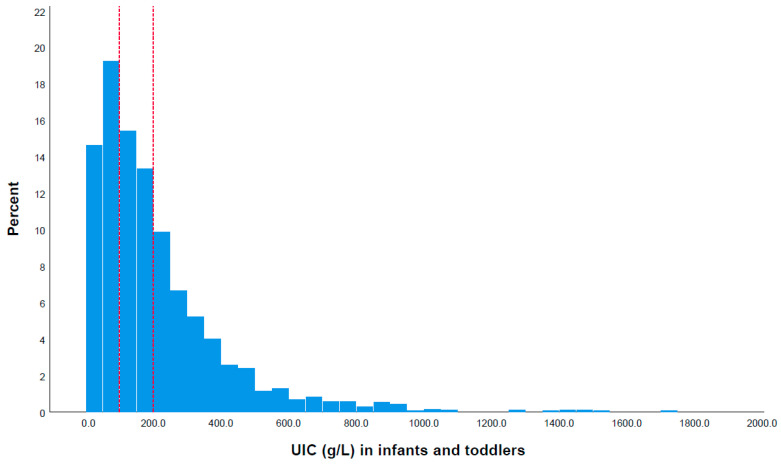
Distribution of urinary iodine concentration (UIC) among infants and toddlers aged 6–24 months: 33.8% had a UIC below 100 μg/L, 28.7% had a UIC between 100 and 199 μg/L, 16.5% had a UIC between 200 and 299 μg/L, and 21.0% had a UIC above 300 μg/L (*n* = 2282). The left and right red dotted lines represent the levels of UIC at 100 μg/L and 199 μg/L, respectively.

**Figure 2:**
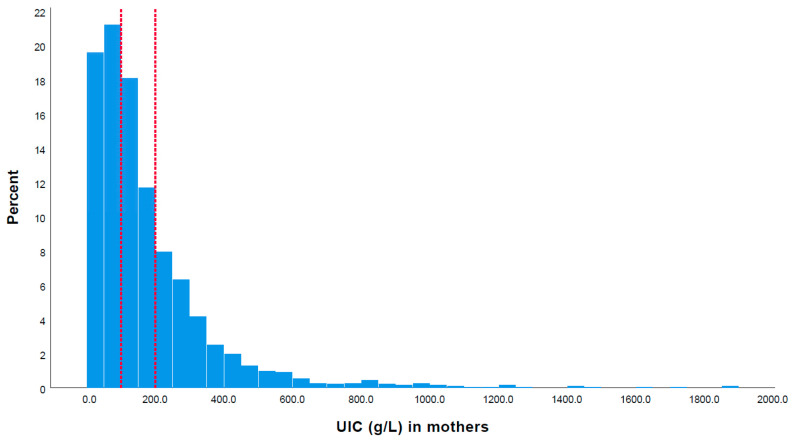
Distribution of urinary iodine concentration (UIC) among mothers: 40.8% had a UIC below 100 μg/L, 29.8% had a UIC between 100 and 199 μg/L, 14.2% had a UIC between 200 and 299 μg/L, and 15.2% had a UIC above 300 μg/L (*n* = 2282). The left and right red dotted lines represent the levels of UIC at 100 μg/L and 199 μg/L, respectively.

Additionally, the median UIC of the children was higher than that of the mothers (*p* > 0.001). As shown in Figure 1, 33.8% of the children had a UIC below 100 μg/L, 28.7% had a UIC between 100 and 199 μg/L, 16.5% had a UIC between 200 and 299 μg/L, and 21.1% had a UIC above 300 μg/L (*n* = 2282). Among the mothers, 40.8% had a UIC below 100 μg/L, 29.8% had a UIC between 100 and 199 μg/L, 14.2% had a UIC between 200 and 299 μg/L, and 15.2% had a UIC above 300 μg/L (*n* = 2282) (Figure 2).

### 3.3. The Relationship between Children’s UIC and Feeding Behaviors and the Frequency of Intake of Iodine-Rich Foods

Of the 2282 infants and toddlers included in the study, the percentages of young children who received breast milk, formula milk, and complementary food (+BM + FM + CF), breast milk and complementary food but no formula milk (+BM − FM + CF), no breast milk but formula milk and complementary food (−BM + FM + CF), and neither breast milk nor formula milk but only complementary food (−BM − FM + CF) were 16.0%, 9.7%, 73.4%, and 0.8%, respectively. About three-fourths of infants and toddlers did not receive any breast milk at the time of sampling. Infants and toddlers who received breast milk 1–3 times per day and ≥4 times per day accounted for 13.7% and 12.1%, respectively. Exclusive breastfeeding for at least 6 months accounted for 55.8%. Only 63.5% of the young children were initially introduced to breast milk. The percentage of infants and toddlers consuming less than 500 mL of formula milk per day and 500 mL or more per day in the last week were 47.4% and 52.5%, respectively. The median (p25–p75) UICs were 146.1 μg/L (71.5–249.5 μg/L) and 156.2 μg/L (79.7–277.9 μg/L), respectively. Infants and toddlers who consumed 500 mL of formula milk per day or above had a higher UIC compared with those who consumed less than 500 mL of formula milk per day (*p* = 0.026). Most of the young children (72.5%) consumed regular complementary food four times or more per day. A total of 35.0% of the young children consumed iodized salt, and 18.7% had been introduced to condiments. Only 1.9% of them used iodine-containing supplements. The UICs among the infants and toddlers across different feeding behaviors are shown in Table 2. No significant differences in UIC were found across different feeding practices, daily frequencies of breastfeeding, duration of exclusive breastfeeding, type of milk feeds first introduced, daily frequencies of regular complementary food, and the use of iodized salt, condiments and iodine-containing supplements.

Table 3 presents the frequency of intake of iodine-rich foods among infants and toddlers in the last month. Eggs and infant formula were commonly consumed among young children, but 3.5% and 12.1% were never or rarely given eggs or infant formula, respectively. About two-thirds of the children were given fish 1–3 times per week or more frequently, while about one-third of the children consumed livestock and poultry meat at least once per day. A total of 66.4% and 78.9 were rarely or never given milk or other milk or cheese. Laver, seaweed, dried small shrimp, and dried mussel were not commonly consumed, with less than one time per month by 71.2%, 85.3%, 68.7%, and 93.2% of individuals. There were no statistically significant differences in the UIC among the different consumption frequencies for any of the foods.

### 3.4. Correlation between the Iodine Status of Infants and Toddlers and Their Mothers

**Figure 3:**
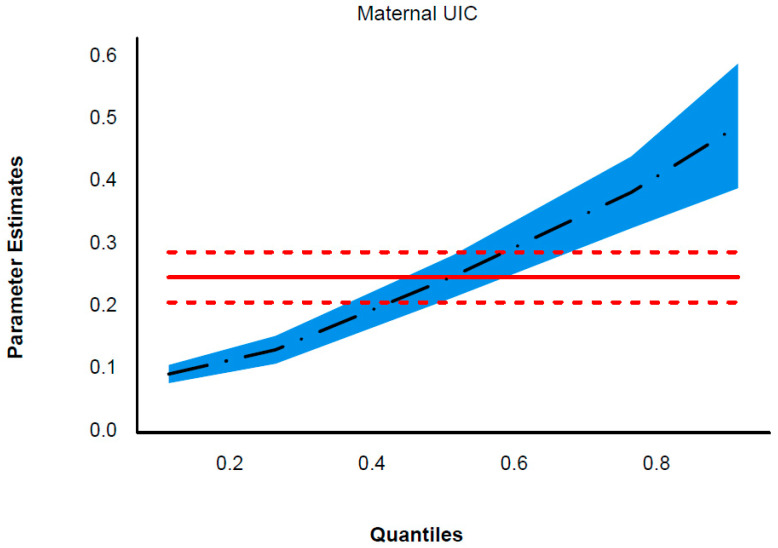
Plot of the maternal UIC effect on quantiles from multivariable simultaneous quantile regression (black dots) and their associated 95% confidence intervals (blue shaded regions). Note: The quantiles used are 0.1, 0.25, 0.5, 0.75, and 0.9, represented by the dots (.) in the plots. The solid red lines are the ordinary least-squares regression lines with their 95% confidence intervals (dashed red lines).

A positive correlation was observed between infants and toddlers’ UIC and maternal UIC as continuous variables (*r_s_* = 0.285, *p* < 0.001) (Table 1). Table 4 presents the coefficients from the quantile regression analysis at different conditional points in the children’s UIC distribution (0.1, 0.25, 0.5, 0.75, 0.9). The variables, including maternal UIC, maternal BMI, maternal age, maternal educational level, children’s gender, children’s age, household income, children’s salt category, iodine supplement usage, feeding practice, and daily consumption of formula milk in the last week, showed differential effects depending on whether the modeling was performed at the conditional quantiles of the children’s UIC distribution. Quantile regression at 0.1, 0.25, 0.5, 0.75, and 0.9 revealed a statistically significant positive association between maternal UIC and the children’s UIC (q0.10: β 0.094, 95% CI 0.079, 0.108; q0.25: β 0.133, 95% CI 0.110, 0.155; q0.50: β 0.251, 95% CI 0.217, 0.284; q0.75: β 0.385, 95% CI 0.327, 0.442; q0.90: β 0.491, 95% CI 0.392, 0.591). At the 0.10 quantile, there was a significant correlation between the maternal education level, salt category, iodine-containing supplement use, and daily consumption of formula milk in the last week and the UIC levels of children. Children whose mother’s education level was high school or junior college had lower UIC levels compared with those whose mother’s education level was university or higher (q0.10: β −9.213, 95% CI −15.689, −2.738). Similarly, children consuming iodized salt had higher UIC levels than those who never consumed salt or who consumed non-iodine salt (q0.10: β 6.295, 95% CI 0.188, 12.402), while children with no iodine-containing supplement use had lower UIC levels than those using iodine-containing supplements (q0.10: β −25.963, 95% CI −46.646, −5.279). Meanwhile, children with a daily consumption of formula milk in the last week of <500 mL per day had lower UIC levels than those consuming ≥500 mL per day (q0.10: β −9.427, 95% CI −15.828, −3.027). At the 0.90 quantile, children’s age and feeding practice had a statistically significant association with the UIC levels in the children. A significant negative correlation was observed between children’s age and their UIC levels (q0.90: β −20.642, 95% CI −36.620, −4.664). Additionally, children who consumed breast milk, formula milk, and complementary food, as well as those who consumed formula milk and complementary food without breast milk had lower UIC levels compared with children who consumed neither breast milk nor formula milk but only complementary food (q0.90: β −86.285, 95% CI −168.254, −4.316; β −97.740, 95% CI −153.992, −41.488). Figure 3 presents a plot of estimated coefficients for maternal UICs across the conditional quantiles of the children’s UIC.

## 4. Discussion

Our study provides new and extensive data on the iodine status of young children and mothers in Shanghai from 6 to 23 months after birth. We found that children, lactating women, and weaning women all had a sufficient iodine status according to the WHO criteria, with median (p25–p75) UICs of 152.1 μg/L (75.8–268.3 μg/L), 121.3 μg/L (68.1–206.4 g/L), and 123.4 (58.4–227.2 g/L), respectively. Similar results were also reported in a small-scale study conducted in Shanghai among 257 lactating women and their infants between May 2018 and May 2019 [21]. The median (p25–p75) UIC of lactating women in that study was 110.0 μg/L (65.8–171.4 μg/L), while the median (p25–p75) UIC of infants was 212.7 μg/L (142.1–320.6 μg/L), both indicating iodine sufficiency [21]. A Norwegian study involving toddlers aged 18 months found a median (p25–p75) UIC of 129 μg/L (81–190 μg/L) [30], which is consistent with our findings. Several reports from countries with more-than-adequate or excessive iodine intakes have also shown similar median UIC values in infants (2 years old) compared to our study [31,32,33,34]. Although there is no established threshold for excessive iodine intake during infancy (0–2 years old) and lactation, median UICs of < 100 and ≥ 300 μg/L are considered deficient and excessive, respectively, in children older than 6 years [13]. In our study, however, it is concerning that 33.8% of children and 40.8% of mothers had a UIC below 100 μg/L, suggesting that they may be at risk of ID. Furthermore, 21.0% of the children and 15.2% of the mothers had a UIC above 300 μg/L, indicating potential iodine overload [35]. Interestingly, we observed significantly higher UICs in the children compared with the mothers, which is consistent with a study conducted in Henan Province, China. A possible reason may be that the thyroid and renal functions in children are immature, and therefore excess iodine cannot be retained [36].

The majority (90%) of young children in our study had already received formula milk, and all of the children had partially received complementary food, including regular meals or snacks, at the time of sampling. In our study, no significant differences were observed in the median UIC values in infants and toddlers among four different feeding practices (*p* = 0.350). A study conducted in Boston among 95 infants less than 3 months of age found that the UICs of infants who were exclusively breastfed, formula-fed, and mixed fed were 203.5 μg/L, 182.5 μg/L, and 197.8 μg/L, respectively. There were no statistically significant differences between the three groups (*p* = 0.88) [37]. Another research study conducted in Norway, which included 57 infants under the age of 2 years with cow’s milk protein allergy found that the median UIC of the partially breastfed and weaned groups were 172 μg/L and 175 μg/L, respectively, compared with 86 μg/L in the exclusively breastfed children. However, the difference was not statistically significant between the groups (*p* = 0.07) [38]. These data are similar to our findings, suggesting no difference in iodine status across different feeding practices. However, Andersson et al. conducted a study on iodine nutrition among 3-to 4-day-old and 6-to 12 -month-old infants in Switzerland and found that infants who received infant formula (exclusively or in addition to breastmilk) had significantly higher UICs than those who were exclusively breastfed (109 vs. 70 μg /L; *p* < 0.01) [39]. Similarly, a study conducted in New Zealand from June 2016 to December 2017 found that infants aged 6 months who were partially breastfed had a higher UIC (147 μg/L) than those who were exclusively breastfed (80 μg/L) [40]. These data are in contrast to our findings. Exclusively breastfed infants are highly reliant on the iodine content in breast milk, which is dependent on the iodine status of breastfeeding mothers [38]. Mulrine’s research found a substantial decrease in breast-milk iodine concentration (BMIC) during the first 6 months postpartum [41]. Ying Jin’s study also showed a significant reduction in BMIC from 3 months postpartum to 6 months postpartum to 12 months postpartum in women who were breastfeeding [40]. Furthermore, a US diet study found that 90% of the iodine intake in infants older than 6 months was provided by infant formula/foods and dairy products [42]. In our study, all children were at least 6 months old and received complementary food, with approximately 90% receiving formula milk; thus, they had various sources of iodine, such as complementary foods or infant formula, which offered a wide range of acceptable iodine content. Therefore, they may have acquired more iodine compared with exclusively breastfed infants who rely solely on breast milk for thyroid function. Consequently, the urinary iodine levels of children with different feeding practices in our study may not exhibit significant variation.

We did find a significant difference in UIC across different levels of daily consumption of formula milk in the last week (*p* = 0.026). Children who consumed less than 500 mL of formula milk per day had lower UIC values compared with children who consumed 500 mL or more per day. A survey conducted in New Zealand among 6-to-24-month-old children found that energy from infant formula was significantly associated with UIC in children [43]. Other studies have also reported a correlation between UIC and the use of infant formula [39,43], which is consistent with our findings. This may be due to the fact that infant formula milk is enriched with iodine [44,45].

However, we did not find any correlation between UIC and other feeding behaviors, including daily frequencies of breastfeeding, duration of exclusive breastfeeding, type of milk feeds first introduced, daily consumption of formula milk in the last week, daily frequencies of regular complementary food, addition of salt to the infants’ food, condiments already introduced, and iodine nutrient supplement use.

Only 35.0% of infants and toddlers in our study had the addition of iodized salt to their food. Andersson et al. suggested that iodized salt contributes little dietary iodine for weaning infants [39]. In our study, we did not find a significant difference in UIC among children who consumed iodized salt compared to those who did not consume salt or who consumed non-iodized salt.

Adequate iodine intake is important for the growth and development of the fetus and infant. Some studies have reported a connection between the use of iodized salt and increased weight and arm circumference in infants, particularly in the second year of life [46]. Jin Yang, et al. also suggested that maternal iodine status during lactation may be related to infant anthropometric indices [36]. However, there is limited research on the association between infant UIC and anthropometric measurements. While our study was not specifically designed to estimate the prevalence of wasting, underweight, and overweight in Shanghai infants and toddlers, our analysis of anthropometric measurements in the sample provided valuable insights. Our data indicated some degree of malnutrition among the studied children. Specifically, the prevalence of wasting, underweight, and overweight among children was found to be 0.7%, 0.9%, and 7.1%, respectively. Our findings suggest a relatively better situation regarding wasting and underweight but a worse scenario concerning overweight compared with a study conducted among 17,193 6–23-month-old children in rural China, where the prevalence of wasting, underweight, and overweight were 2.0%, 2.1%, and 3.0%, respectively [47]. In our study, we found a lack of significant associations between children’s iodine status and anthropometric measurements. Previous studies have often focused on the associations between maternal iodine intake from supplements during pregnancy and lactation and child neurodevelopment. Some results have shown no evidence of a protective effect of iodine supplementation during pregnancy on child neurodevelopment [48]. However, less is known about the potential consequences on children’s growth and development and of the use of iodine supplements in young children. In our study, we found that there was no statistically significant correlation between iodine-containing supplement use and UIC in children (Table 2).

In our study, we found that eggs and infant formula were the two most frequently consumed iodine-rich foods. However, we did not observe any statistically significant differences in UIC based on the frequency of consumption of various iodine-rich foods. A prospective population-based cohort study conducted in Norway between 2011 and 2014 among 777 toddlers aged 18 months also found no substantially or statistically significant differences in UIC between different consumption categories for any of the foods [30]. This outcome aligns with our findings. One possible reason for this lack of association could be that we only recorded the frequencies of food intake and not the quantities consumed, which was a limitation of the dietary data.

In our Shanghai cohort, we found a positive correlation between children’s iodine status and maternal iodine status at every conditional quantile. Previous studies have indicated that infant UIC can be influenced by their mothers’. Pantea Nazeri, et al. suggested that a positive association between urinary iodine status in mothers and exclusively breastfed infants could be expected. Their study found that the UIC value of newborns was associated with maternal urinary iodine (β = 0.191, *p* = 0.048) [49]. Similarly, a study conducted in the city of Zhengzhou in China found a significant positive correlation between the UIC of breastfeeding mothers and infants (*r_s_* = 0.104, *p* = 0.004) [50]. A systematic review also concluded that BMIC serves as a biomarker of iodine status in children under 2 years of age [51]; our study aligns with these previous findings. The reason for this correlation may be that both children and mothers derive their iodine from the same food sources.

The main strength of our present study is the simultaneous assessment of the iodine status among infants and toddlers in Shanghai with different feeding behaviors, as well as among their mothers. This allows the research findings to possess regional characteristics and representativeness, providing important information about the iodine nutrition status of young children aged 6–23 months and their mothers in Shanghai. Furthermore, this study found a positive correlation between children’s iodine status and their mothers’ iodine status. This finding further emphasizes the importance of the mother–child association and reminds us to pay attention to mothers’ iodine intake when improving children’s iodine nutrition status. However, limitations of our study include the lack of data on factors related to maternal and young children’s iodine status, such as BMIC. The random UIC in lactating women can be influenced by maternal fluid intake, so 24 h urinary iodine excretion may provide a more accurate evaluation of iodine status in lactating women [34,52].

## 5. Conclusions

In conclusion, the findings from this study suggest that young children aged 6–23 months and their mothers living in Shanghai, China, have sufficient iodine levels. The median UIC for young children, lactating mothers, and weaning mothers was 152.1 μg/L, 121.3 μg/L, and 123.4 μg/L, respectively. However, a large minority of children and mothers may be at risk of ID. Young children who consumed ≥500 mL per day of formula milk in the last week had a significantly higher median UIC compared with those who consumed <500 mL per day of formula milk. Additionally, there was a positive correlation between the UIC values of young children and their mothers.

## Figures and Tables

**Table 1:** Urinary iodine concentration values of the infants and toddlers by demographic characteristics of the mothers and children.

Characteristics	*n*	%	Mean ± SD or Median (P25–P75)	Children’s UIC (μg/L)			*r_s_*	*p*-Value
				Median	P25	P75		
Infants and toddlers	2282	-	-	152.1	75.8	268.3		
Gender	2282	-	-					
Boys	1203	52.7	-	148.7	72.8	261.9		0.213
Girls	1079	47.3	-	155.3	79.7	279.5		
Age group	2281	-	12.9 ± 1.3					0.897
6–11 months	1591	69.7	-	152.1	74.2	273.9		
12–23 months	69.0	30.2	-	152.1	83.6	250.8		
Gestational age at birth, (completed weeks)	2233	-	39 (38–40)				−0.002	0.930
Weight-for-length/height, z-score	2244	-	0.6 ± 0.982				0.010	0.642
<−2 (wasted)	15	0.7	−2.73 ± 0.80	181.5	65.7	234.4		0.941
>2 (overweight)	161	7.1	2.71 ± 0.69	151.0	81.3	288.2		
BMI-for-age, z-score	2233	-	0.59 ± 1.02				0.004	0.838
<−2 (underweight)	20	0.9	−2.66 ± 0.62	187.6	71.1	250.1		0.957
>2 (overweight)	163	7.1	2.82 ± 0.71	154.6	75.2	244.1		
Total annual household income (CNY)	2272							
Low (≤149,000)	592	25.9		156.5	88.0	268.3		0.132
Medium (150,000–299,000)	1171	51.3		147.5	70.8	263.9		
High (≥300,000)	509	22.3		155.9	79.7	276.9		
Mothers								
Age (years)	2282		30.5 ± 4.4					
<29	1033	45.3		152.6	76.5	268.2		0.784
30–34	797	34.9		149.9	74.2	260.5		
35–39	382	16.7		156.0	78.4	279.6		
≥40	70	3.1		149.8	59.6	272.8		
Maternal BMI (kg/m^2^)	2254		22.2 ± 3.0				−0.009	0.656
<18.5	180	7.9		161.0	98.7	255.1		0.322
18.5–24.9	1721	75.4		150.7	73.0	267.2		
25–29.9	318	13.9		160.2	83.8	278.0		
≥30	35	1.5		155.9	57.4	247.1		
Educational level	2282	-	-					
Junior high school or below (≤9 years)	361	15.8		157.7	81.3	262.5		0.920
High school or junior college (10–15 years)	1035	45.4		152.9	74.3	274.4		
University or higher(≥16 years)	886	38.8		149.7	75.2	265.8		
Birth parity	2282							
Primiparous	1398	61.3		152.3	73.3	265.8		0.908
Multiparous	884	38.7		152.0	80.3	273.4		
Smoking status	2282							
No	2260	99.0		151.9	75.3	267.2		0.227
Yes	22	1.0		177.7	110.4	296.7		
Alcohol intake during lactation	2282							
Yes	13	0.6		131.8	117.4	270.2		0.757
No	2269	99.4		152.4	75.5	267.5		
Maternal UIC (μg/L)	2282	-	123.1 (62.3, 220.9)				0.285	<0.001
UIC of lactating women	588	25.8	121.3 (68.1, 206.4)					
UIC of weaning women	1694	74.2	123.4 (58.4, 227.2)					

UIC, urinary iodine concentration; WLZ, weight-for-length/height z-score; BAZ, BMI-for-age z-score; P25, 25th percentile; P75, 75th percentile.

**Table 2:** Children’s UIC values in relation to their feeding behaviors.

Feeding Behaviors	*n*	%	Children’s UIC (μg/L)			
			Median	P25th	P75th	*p*-Value
Feeding practice	2282					
+BM + FM + CF	366	16.0	159.5	80.9	295.4	0.350
+BM − FM + CF	222	9.7	146.6	73.9	244.9	
−BM + FM + CF	1676	73.4	150.6	75.2	266.8	
−BM − FM + CF	18	0.8	182.6	52.1	340.5	
Daily frequencies of breastfeeding	2282					
0 times per day	1694	74.2	150.9	75.2	267.0	0.650
1–3 times per day	312	13.7	158.8	79.3	282.0	
≥4 times per day	276	12.1	153.9	75.9	262.5	
Duration of exclusive breastfeeding	2279					
0–5 months	1005	44.0	154.2	73.8	277.9	0.570
6 months	614	26.9	150.6	79.6	270.5	
≥7 months	660	28.9	150.4	76.9	253.5	
Type of milk feeds first introduced	2276					
Breast milk	1449	63.5	153.6	75.9	270.2	0.522
Formula milk	827	36.2	149.5	75.3	262.5	
Daily consumption of formula milk in the last week	2279					
<500 mL per day	1082	47.4	146.1	71.5	249.5	0.026
≥500 mL per day	1195	52.5	156.2	79.7	277.9	
Daily frequencies of regular complementary food	2282					
1–3 times per day	628	27.5	160.1	82.2	280.0	0.071
≥4 times per day	1654	72.5	148.1	73.9	263.5	
Addition of salt to the infants’ food	2282					
Iodized salt	798	35.0	156.1	82.0	276.4	0.223
Never or non-iodized salt	1484	65.0	149.7	73.9	265.3	
Condiments already introduced	2282					
Yes	427	18.7	151.9	80.0	272.7	0.690
No	1855	81.3	152.1	75.2	267.0	
Iodine-containing supplements used	2282					
No	2238	98.1	151.9	75.2	268.2	0.402
Yes	44	1.9	176.1	106.7	262.6	

+BM + FM + CF, children receiving breast milk, formula milk, and complementary food; +BM − FM + CF, children receiving breast milk and complementary food but no formula milk; −BM + FM + CF, children receiving no breast milk but formula milk and complementary food, −BM − FM + CF, children receiving neither breast milk nor formula milk, only complementary food. UIC, urinary iodine concentration; P25, 25th percentile; P75, 75th percentile.

**Table 3:** Frequency of intake (times/week) of iodine-rich foods among Shanghai infants and toddlers.

Iodine-Rich Foods	*n*	At Least 1 Time per Day	1–3 Times per Week	1–3 Times per Month	Never/Rarely
Eggs	2215	1064 (70.3)	471 (20.6)	61 (2.7)	79 (3.5)
Infant formula	2282	1890 (82.8)	67 (2.9)	49 (2.1)	276 (12.1)
Fish	2215	227 (9.9)	1356 (59.4)	454 (19.9)	178 (7.8)
Livestock and poultry meat	2215	780 (34.2)	1046 (45.8)	251 (11.0)	138 (6.0)
Milk or other animal milk	2282	397 (17.4)	203 (8.9)	167 (7.3)	1515 (66.4)
Cheese	2214	60 (2.6)	109 (4.8)	244 (10.7)	1801 (78.9)
Laver	2282	24 (1.1)	228 (10.0)	405 (17.7)	1625 (71.2)
Seaweed	2282	7 (0.3)	83 (3.6)	246 (10.8)	1946 (85.3)
Dried small shrimp	2282	32 (1.4)	260 (11.4)	423 (18.5)	1567 (68.7)
Dried mussel	2282	5 (0.2)	24 (1.1)	127 (5.6)	2126 (93.2)

The difference in children’s UIC between the four groups was determined by the Kruskal–Wallis test; all *p* ≥ 0.05.

**Table 4:** Results of multivariable simultaneous quantile regression analysis for infants and toddlers’ UIC.

Variables	Coefficients (95%CI)				
	0.10 Quantile	0.25 Quantile	0.5 Quantile	0.75 Quantile	0.90 Quantile
Maternal UIC	0.094 (0.079, 0.108) ***	0.133 (0.110, 0.155) ***	0.251 (0.217, 0.284) ***	0.385 (0.327, 0.442) ***	0.491 (0.392, 0.591) ***
Maternal BMI	0.124 (−0.856, 1.104)	−0.562 (−2.072, 0.948)	−0.283 (−2.582, 2.016)	−1.286 (−5.186, 2.613)	−6.004 (−12.781, 0.773)
Maternal age (years)					
<29	6.143 (−10.804, 23.091)	6.997 (−19.104, 33.098)	−10.608 (−50.352, 29.135)	−5.590 (−72.998, 61.818)	39.049 (−78.096, 156.194)
30–34	8.997 (−8.095, 26.088)	9.745 (−16.577, 36.067)	−2.033 (−42.113, 38.047)	−5.627 (−73.606, 62.352)	−3.414 (−121.552, 114.724)
38–39	4.855 (−12.910, 22.620)	12.285 (−15.074, 39.645)	−0.870 (−42.530, 40.790)	30.279 (−40.380, 100.937)	74.326 (−48.468, 197.121)
≥40	ref				
Maternal education level (years)					
Junior high school or below (≤9)	−5.702 (−14.949, 3.546)	−10.551 (−24.793, 3.691)	−6.871 (−28.558, 14.815)	−10.628 (−47.409, 26.154)	−11.576 (−75.497, 52.345)
High school or junior college (10–15)	−9.213 (−15.689, −2.738) **	−8.720 (−18.692, 1.252)	8.105 (−7.080, 23.289)	−0.110 (−25.864, 25.644)	−6.704 (−51.461, 38.052)
University or higher (≥16)	ref				
House Income (CNY)					
Low (≤149,000)	3.687 (−5.117, 12.490)	8.202 (−5.356, 21.760)	−1.678 (−22.323, 18.966)	6.631 (−28.384, 41.654)	−33.497 (−94.347, 27.353)
Medium (150,000–299,000)	−5.785 (−13.131, 1.560)	−3.207 (−14.520, 8.106)	−7.906 (−25.132, 9.319)	−0.342 (−29.558, 28.874)	−11.577 (−62.350, 39.196)
High (≥300,000)	ref				
Children’s age (months)	−0.207 (−2.519, 2.104)	−1.502 (−5.062, 2.058)	−1.535 (−6.956, 3.886)	−11.998 (−21.193, −2.804) *	−20.642 (−36.620, −4.664) *
Children’s gender					
Male	−0.229 (−5.978, 5.521)	−0.157 (−9.011, 8.697)	−11.090 (−24.572, 2.392)	−5.195 (−28.062, 17.672)	−4.979 (−44.717, 34.760)
Female	ref				
Salt category					
Iodized salt	6.295 (0.188, 12.402) *	3.998 (−5.408, 13.403)	7.200 (−7.121, 21.521)	15.421 (−8.869, 39.710)	20.222 (−21.990, 62.434)
Never or non-iodized salt	ref				
Iodine-containing supplement use					
No	−25.963 (−46.646,−5.279) *	−29.007 (−60.862, 2.848)	−23.532 (−72.037, 24.973)	−28.491 (−110.759, 53.777)	47.466 (−95.504, 190.435)
Yes	ref				
Feeding practice					
+BM + FM + CF	0.671(−11.188, 12.530)	3.917 (−14.346, 22.181)	−8.158 (−35.968, 19.651)	−20.887 (−68.054, 26.280)	−86.285 (−168.254, −4.316) *
+BM − FM + CF	3.854(−29.955, 37.663)	−14.831 (−66.900, 37.238)	33.075 (−46.209, 112.359)	48.191 (−86.280, 182.663)	−32.062 (−265.754, 201.631)
−BM + FM + CF	−3.124(−11.262, 5.014)	−4.045 (−16.579, 8.488)	−9.619 (−28.704, 9.466)	−16.419 (−48.788, 15.950)	−97.740 (−153.992, −41.488) **
−BM − FM + CF	ref				
Daily consumption of formula milk in the last week					
<500 mL per day	−9.427(−15.828, −3.027) **	−5.225 (−15.082, 4.632)	−6.899 (−21.908, 8.110)	−19.937 (−45.394, 5.519)	−8.775 (−53.015, 35.465)
≥500 mL per day	ref				

CI, confidence interval; ref, reference category; *: *p*-value < 0.05; **: *p*-value< 0.01; ***: *p*-value < 0.001.

## Data Availability

The data used to support the findings of the current study are available from the corresponding author. The data are not publicly available due to policy restrictions.
